# Application of the Multi-line Tension-Reduction Technique in the Surgical Correction of C-shaped Deviations of the Nasal Septum

**DOI:** 10.7759/cureus.82769

**Published:** 2025-04-22

**Authors:** Yan Li, Gang Y Jin

**Affiliations:** 1 Otorhinolaryngology, Shanghai Yida Hospital, Shanghai University, Shanghai, China; 2 Otorhinolaryngology, Xianghe County People's Hospital, Hebei Medical University, Langfang, CHN

**Keywords:** complications, correction effect, c-shaped deviation of nasal septum, multi-line tension reduction surgery, postoperative recovery

## Abstract

Objective

This study aims to evaluate the efficacy of the multi-line tension-reduction technique in the surgical correction of a C-shaped nasal septum deviation.

Methods

This study included patients diagnosed with a C-shaped nasal septum deviation who underwent nasal septum correction surgery. Participants were divided into two groups: an observation group, in which the multi-line tension-reduction technique was applied, and a control group, in which traditional surgical methods were used. The following parameters were compared between the two groups: operation time, intraoperative blood loss, postoperative pain intensity (assessed using the visual analog scale (VAS)), improvement in nasal ventilation (measured using a nasal resistance meter), and complication rate.

Results

In the observation group, the mean operation time was 35.22 ± 5.48 minutes and the mean intraoperative blood loss was 10.53 ± 4.07 mL, both of which were significantly lower than those in the control group (t = 7.84, 16.215; P < 0.05). The postoperative pain intensity, as assessed by VAS scores at 24, 48, and 72 hours, was 5.23 ± 1.23, 2.48 ± 1.09, and 0.68 ± 0.76 points, respectively, all of which were significantly lower than those in the control group (t = 8.11, 11.80, 8.383; P < 0.05). The complication rate in the observation group was 2.5%, which was significantly lower than that in the control group (χ² = 3.97; P < 0.05). Nasal ventilation resistance values at 1, 3, 6, and 12 months post-operation were 0.87 ± 0.12, 0.66 ± 0.16, 0.64 ± 0.13, and 0.61 ± 0.14, respectively, all of which were significantly lower than those in the control group (t = 9.221, 9.477, 8.923, 7.212; P < 0.05). These findings indicate that the multi-line tension-reduction technique significantly outperformed traditional surgical methods in terms of operation time, intraoperative blood loss, postoperative pain intensity, nasal ventilation improvement, and complication rate.

Conclusion

The application of the multi-line tension-reduction technique in the surgical correction of a C-shaped nasal septum deviation exhibits substantial advantages such as reduced operation time, minimized intraoperative blood loss, attenuated postoperative pain, improved recovery of nasal ventilation function, and a significantly lower complication rate.

## Introduction

Nasal septum deviation is a common anatomical abnormality of the nose, among which the C-shaped deviation is more common. A nasal septum deviation, a condition where the nasal septum is crooked or off-center, can result in nasal ventilation disorders, epistaxis, and headaches. These symptoms significantly impair the patient's quality of life, as they may lead to chronic nasal congestion, persistent headaches, and recurrent nosebleeds. Septoplasty, a surgical procedure aimed at correcting abnormal anatomical structures, is the primary treatment for nasal septum deviation. The traditional surgical methods have some shortcomings, and the multi-line tension-relieving technique, as an improved surgical method, may be of great significance to improve the surgical effect [1-3].

## Materials and methods

General information

Eighty patients diagnosed with C-shaped deviations of the nasal septum via nasal endoscopy underwent corrective surgery at our hospital between April 2022 and April 2024. The success rate of such surgeries is generally high, with reported rates ranging from 90% to 99%, depending on factors such as the severity of the deviation, postoperative care, and the patient's overall health [4-5]. There were 40 patients in the observation group, including 34 males and 6 females, aged from 22 to 72 years, with an average age of 37.28±12.62 years. There were 40 patients in the control group, including 34 male patients and 6 female patients, aged 21-71 years, with an average age of 40.75±12.26 years. There was no significant difference in age, gender, and other general data between the two groups (P > 0.05), with comparability. This study was approved by the ethics committee of our hospital (20220301), and all patients were aware and agreed.

Inclusion and exclusion criteria

Inclusion Criteria

Patients must meet the diagnostic criteria for a C-shaped deviation of the nasal septum, which includes symptoms, such as unilateral or bilateral nasal congestion, headache, and epistaxis, due to the convexity of the deviation, and have complete medical records available for review. Family members must be informed and have consented to the research.

Exclusion Criteria

Patients with nasal tumors or symptoms complicated by nasal polyps and sinusitis were excluded. Patients with a previous history of nasal surgery were also excluded.

Surgical methods

Control Group (Traditional Surgical Method)

Patients were placed in the supine position and underwent conventional submucosal resection of the nasal septum after local or general anesthesia. The perichondrium and periosteum were separated along one side of the nasal septum mucosa, and the deviated nasal septal cartilage and bone were exposed. According to the deviation, part of the cartilage and bone were removed, the mucosa was repositioned, and the nasal cavity was tamponaded with an absorbable and expansive sponge for 48 hours to stop bleeding [4-5].

Observation Group

**Figure 1:**
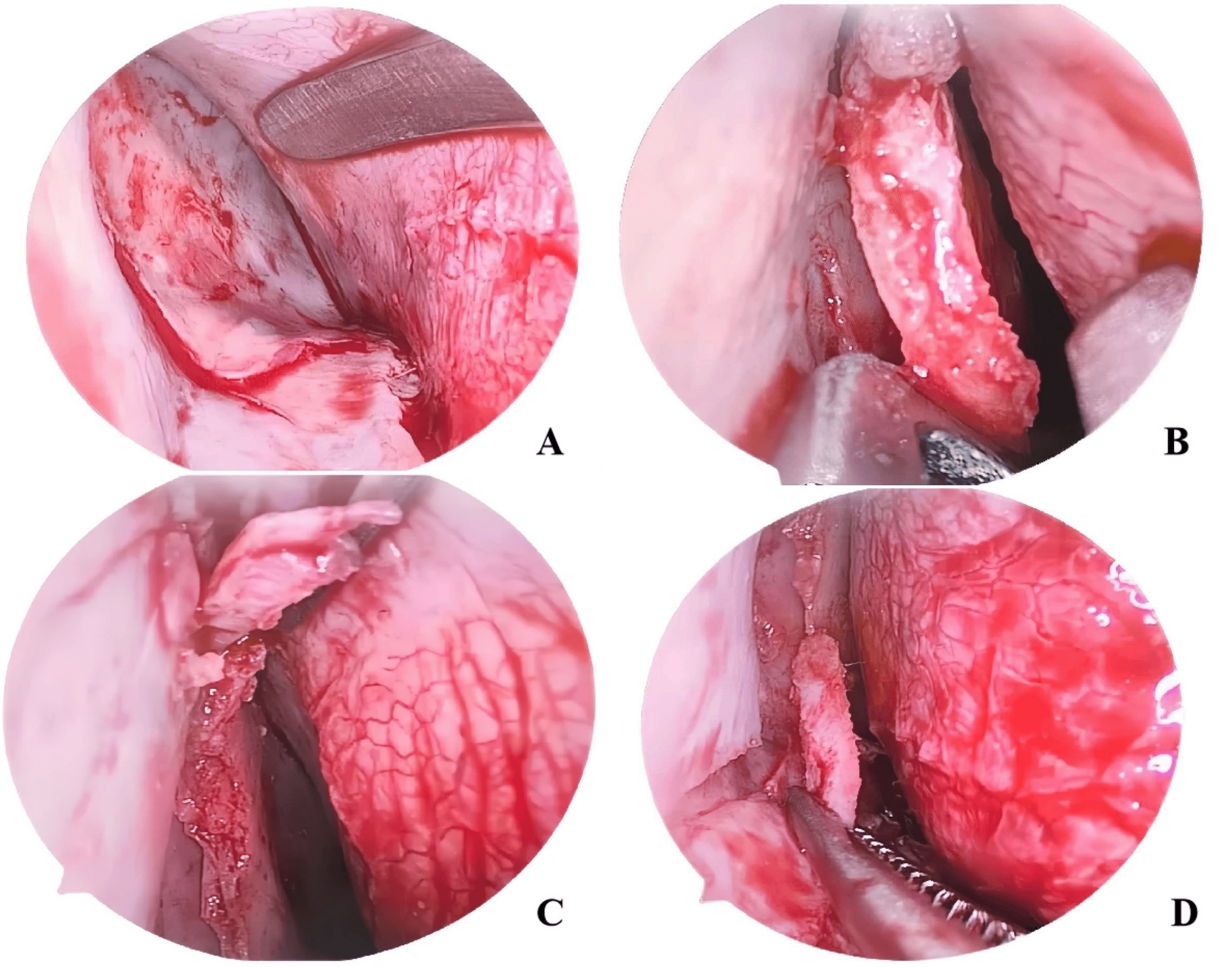
Multi-line tension-relieving method 1-A illustrates the perichondrium and periosteum of the nasal septum on the separated side. 1-B depicts a 1.5 mm longitudinal resection at the anterior end of the nasal septum. 1-C shows a 1.5 mm longitudinal resection at the posterior end of the nasal septum. 1-D presents a 3 mm horizontal resection at the lower end of the nasal septum.

In the observation group, employing the multi-line tension-relieving method, an incision was initially made on the mucosa of the deviated convex surface of the nasal septum. Subsequently, the submucosal tissue was gently dissected to expose the contralateral nasal septum mucosa. To release the tension of the nasal septum, the deviated nasal septum was gradually restored to a relatively normal position, and the purpose of correcting the deviation was achieved by using multi-line tension-reduction surgery. Finally, the mucosal incision was properly sutured, and the nasal cavity was tamponaded with an absorbable and expansive sponge for 48 hours to stop bleeding. See Figure 1.

Observation indicators

Operation time: Defined as the duration from the initial incision to the completion of the surgical procedure, measured in minutes.

Intraoperative blood loss: Estimated based on the volume of blood collected by the suction device and the amount absorbed by gauze during surgery.

Postoperative pain intensity: The intensity of pain following surgery was assessed using the visual analog scale (VAS), ranging from 0 (no pain) to 10 (severe pain). Evaluations were conducted at 24, 48, and 72 hours post-surgery. Research has demonstrated that comprehensive nursing interventions may facilitate a gradual reduction in VAS scores during this period.

Improvement of nasal ventilation: To evaluate the efficacy of nasal surgeries, nasal resistance was quantified using a nasal resistance meter at baseline and at 1, 3, 6, and 12 months post-surgery. The measurement protocol complied with the reference standard described in the 2010 edition of *Four-Phase Nasal Resistance Measurement: Fundamentals and Practice*, published by the European Rhinology Committee. Nasal resistance levels were classified as follows: normal (<0.75), mild (0.75-1.0), moderate (1.0-1.25), and severe (1.25-1.5) [6].

The incidence of complications in surgical procedures varies significantly, with wound infections being the most common, followed by pulmonary infections, postoperative fistulas, and poor wound healing. These complications are influenced by factors such as patient age, pre-existing conditions, and surgical techniques. Studies indicate that at least half of these complications could be preventable with proper medical care and adherence to surgical protocols.

The occurrence of complications such as nasal septum perforation, nasal adhesion, and bleeding was observed and recorded in the two groups.

Statistical methods

All data were analyzed by SPSS 27.0 statistical software (IBM Corp, Armonk, NY, US). Measurement data were expressed as mean ± standard deviation (Mean ± SD), and a t-test was used for comparison between groups. Count data were expressed as rate (%), and the χ2 test was used for comparison between groups. P < 0.05 was considered statistically significant.

## Results

Duration of surgery

The operation time in the observation group was significantly shorter than that in the control group, with a statistically significant difference (t = 7.84, P < 0.05). See Table 1.

**Table 1:** Comparison of operation time between the two groups ((Mean ± SD), min)

Groups	Duration of surgery
Control group (n=40)	46.08±6.83
Observation group (n=40)	35.22±5.48
t values	7.84
P-value	<0.001

Intraoperative blood loss

The intraoperative blood loss in the observation group was significantly lower than that in the control group, with a statistically significant difference (t = 16.215, P < 0.05). See Table 2.

**Table 2:** Comparison of intraoperative blood loss between the two groups ((Mean ± SD), mln)

Groups	Amount of intraoperative blood loss
Control group (n=40)	29.28±6.08
Study group (n=40)	10.53±4.07
t values	16.215
P-value	<0.001

Degree of postoperative pain

The VAS scores for pain at 24 hours, 48 hours, and 72 hours post-operation were significantly lower in the observation group compared to the control group, indicating reduced pain levels in the observation group. These differences were statistically significant (t = 8.11, 11.80, 8.383, all P < 0.05), suggesting that the intervention had a positive impact on pain management. See Table 3.

**Table 3:** Comparison of postoperative pain scores between the two groups (mean ± SD)

Groups	24 hours after surgery	48 hours after surgery	72 hours after surgery
Control group (n=40)	7.43±1.20	5.55±1.24	2.38±1.03
Study group (n=40)	5.23±1.23	2.48±1.09	0.68±0.76
t values	8.11	11.80	8.383
P-value	<0.001	<0.001	<0.001

Improvement in nasal ventilation

Nasal resistance measurements taken at 1, 3, 6, and 12 months post-operation revealed that nasal ventilation resistance in the observation group was significantly lower than that in the control group, with statistically significant differences (t = 9.221, 9.477, 8.923, 7.212, all P < 0.05). The data presented in Table 4 is critical for understanding the clinical application of nasal resistance testing, as it provides insights into the normal range of nasal resistance values and their deviation, which can indicate various nasal conditions.

**Table 4:** Comparison of postoperative nasal resistance between the two groups (mean ± SD)

Groups	Before surgery	1 month after surgery	3 months after the operation	6 months after surgery	12 months after surgery
Control group (n=40)	1.34±0.13	1.09 ±0.10	0.96±0.12	0.91±0.14	0.85±0.15
Study group (n=40)	1.29±0.12	0.87±0.12	0.66±0.16	0.64±0.13	0.61±0.14
t values	1.953	9.221	9.477	8.923	7.212
P-value	0.54	<0.001	<0.001	<0.001	<0.001

Incidence of complications

The incidence of complications following nasal surgeries can vary depending on the procedure type; for example, endoscopic sinus surgery has a reported complication rate of approximately 1%. The incidence of complications in the observation group was significantly lower than that in the control group, with a statistically significant difference (χ² = 3.97, P < 0.05). See Table 5.

**Table 5:** Comparison of complications between the two groups

Groups	Nasal septum perforation (n)	Nasal septal adhesions (n)	Nasal bleeding (n)	Nasal infection (n)	Total incidence (%)
Control group (n=40)	2	4	0	0	15.0
Study group (n=40)	0	1	0	0	2.5
χ2 value					3.97
P-value					0.04

## Discussion

Advantages of multi-line tension-reduction surgery 

*Reduction in Tissue Damage* 

Compared with traditional surgical methods, multi-line tension-reduction surgery does not necessitate extensive resection of nasal septal cartilage and bone, thereby maximizing the preservation of the normal anatomical structure of the nasal septum. This approach reduces damage to the supporting structures of the nasal septum, helps maintain its stability, and decreases the area of tissue trauma during the operation. Consequently, it leads to shorter operation times and reduced intraoperative blood loss[7-8].

*Reduction in Postoperative Pain* 

Due to minimal tissue damage, the postoperative inflammatory response in the nasal mucosa and surrounding tissues is relatively mild, resulting in a significant reduction in patient-reported postoperative pain. Furthermore, the multi-line tension-reduction technique gradually corrects nasal septal deviation, avoiding mechanical irritation caused by extensive resection or forceful correction, thereby further alleviating pain[9].

Promotion of Nasal Ventilation Function Recovery

Recent technological advancements have enabled more precise adjustments to the nasal septum, significantly enhancing nasal airflow and reducing obstructions. Through multi-line tension reduction, the physiological curvature of the nasal septum can be better restored, minimizing the risk of postoperative nasal septal deviation. This effectively reduces nasal ventilation resistance and improves nasal ventilation function [10].

*Minimization of Complications* 

Proper surgical techniques, as emphasized in studies on nasal septum deviation correction surgery, are essential for minimizing complications. Reasonable tension reduction decreases the likelihood of mucosal contact and adhesion, while precise tension-relieving operations lower the risk of nasal septum perforation. Furthermore, avoiding excessive tissue resection facilitates easier healing of the nasal septal mucosa after surgery [11-12].

Limitations and countermeasures of the multi-line tension-reduction technique 

Although multi-line tension-reduction surgery offers numerous advantages, it imposes high technical demands on surgeons. Surgeons must possess a comprehensive understanding of the nasal septum's anatomical structure and precisely control the magnitude and direction of tension relief. During the initial stages of adopting this technique, additional training and hands-on experience are often required. To address these limitations, surgeons can enhance their skills through participation in specialized professional training programs, observation of surgical procedures via instructional videos, and performing surgeries under the guidance of experienced mentors [13-14].

## Conclusions

The application of the multi-line tension-reduction method in the surgical treatment of C-shaped nasal septum deviations has demonstrated significant improvements in clinical outcomes. This technique not only reduces operation time and intraoperative blood loss but also mitigates postoperative pain, facilitates the recovery of nasal ventilation function, and lowers the incidence of complications. Despite certain technical demands, with the ongoing advancement of medical technology and the increasing experience of surgeons, the multi-line tension-reduction technique is anticipated to become an optimal choice for correcting C-shaped nasal septum deviations, thereby providing enhanced surgical outcomes for patients with nasal septum deviations.
